# Clinical audit to enhance safe practice of skilled birth attendants for the fetus with nuchal cord: evidence from a refugee and migrant cohort

**DOI:** 10.1186/1471-2393-14-76

**Published:** 2014-02-20

**Authors:** Megan Parr, Colley Paw Dabu, Nan San Wai, Paw Si Say, Ma Ner, Nay Win Tun, Aye Min, Mary Ellen Gilder, François H Nosten, Rose McGready

**Affiliations:** 1 Shoklo Malaria Research Unit (SMRU), Mahidol-Oxford Tropical Medicine Research Unit, Faculty of Tropical Medicine, Mahidol University, PO Box 46, Mae Sot, Tak 63110, Thailand; 2 Women’s and Children’s Services, Launceston General Hospital, Launceston, Tasmania, Australia; 3 Mahidol-Oxford Tropical Medicine Research Unit (MORU), Faculty of Tropical Medicine, Mahidol University, Bangkok, Thailand; 4 Centre for Clinical Vaccinology and Tropical Medicine, Nuffield Department of Clinical Medicine, University of Oxford, Churchill Hospital, Oxford, United Kingdom

**Keywords:** Audit cycle, Interprofessional learning, Nuchal cord, Quality improvement, Registered midwife, Skilled birth attendant

## Abstract

**Background:**

Current evidence for optimal management of fetal nuchal cord detected after the head has birthed supports techniques that avoid ligation of the umbilical cord circulation. Routine audit found frequent unsafe management of nuchal cord by skilled birth attendants (SBAs) in migrant and refugee birth centres on the Thai-Burmese border.

**Method:**

The audit cycle was used to enhance safe practice by SBA for the fetus with nuchal cord. In the three birth centres the action phase of the audit cycle was initially carried out by the doctor responsible for the site. Six months later a registered midwife, present six days per week for three months in one birth facility, encouraged SBAs to facilitate birth with an intact umbilical circulation for nuchal cord. Rates of cord ligation before birth were recorded over a 24 month period (1-July-2011 to 30-June-2013) and in-depth interviews and a knowledge survey of the SBAs took place three months after the registered midwife departure.

**Results:**

The proportion of births with nuchal cord ligation declined significantly over the four six monthly quarters from 15.9% (178/1123) before the action phase of the audit cycle; to 11.1% (107/966) during the action phase of the audit cycle with the doctors; to 2.4% (28/1182) with the registered midwife; to 0.9% (9/999) from three to nine months after the departure of the registered midwife, (p < 0.001, linear trend). Significant improvements in safe practice were observed at all three SMRU birth facilities. Knowledge of fetal nuchal cord amongst SBAs was sub-optimal and associated with fear and worry despite improved practice. The support of a registered midwife increased confidence of SBAs.

**Conclusion:**

The audit cycle and registered midwife interprofessional learning for SBAs led to a significant improvement in safe practice for the fetus with nuchal cord. The authors would encourage this type of learning in organizations with birth facilities on the Thai-Burmese border and in other similar resource limited settings with SBAs.

## Background

There is significant economic disparity between Thailand and her surrounding neighbours including Myanmar, Laos and Cambodia. Thailand attracts migrant workers due to the relative stability and employment opportunities. To the west of Thailand is Myanmar, which has been amongst the thirty poorest countries for decades. As a result there are an estimated 1.5-3 million migrants and refugees living and/or working within Thailand [1]. Healthcare for refugees is predominantly implemented by a number of international non-government organizations with regular border wide meetings to co-ordinate care. Migrant health care has lacked organizational support and is problematic due to barriers such as security check-points, financial and language limitations. In resource limited areas of the world the availability of skilled birth attendants (SBAs) during childbirth is a key indicator for Millennium Development Goal 5 as one of the strategies to reduce maternal and neonatal mortality [2]. Refugee and migrant women are a particularly vulnerable group [3,4] and ensuring quality of care of SBAs in such settings a neglected area [5,6].

Shoklo Malaria Research Unit (SMRU) has provided maternity services in Tak Province to the refugee population since 1986 and to the rural migrant population since 1998. SBAs are the primary carers for women birthing. At SMRU SBAs are hired from the local refugee and migrant communities and are salaried workers paid by SMRU. The availability of SBAs, particularly those with experience, has diminished as a result of high population movement, mostly due to relocation of the refugee population to countries of resettlement. Part of the response to this problem was formalization of SBA training in 2010 with a curriculum that includes three months of theory and twelve months of clinical practice. Recruitment to the course was from local communities where literacy remains lower than 60% [7]. The SMRU curriculum and other international non-government organizations health-worker curricula are not formally recognized by Myanmar or Thailand. Three levels of SBAs are recognised at SMRU: assistant, junior and senior. An assistant SBA has successfully completed high school and the SBA formal training course provided by SMRU. Progression to junior and senior positions is achieved by competency based assessment by senior SBAs and by doctors, respectively. Basic English language skills, successful completion of the Advanced Life Support in Obstetrics (ALSO®) course and leadership abilities are required to achieve senior SBA status. Amongst twelve senior SBAs at SMRU, one had a formal tertiary qualification in midwifery from Myanmar while the remainder have completed the SMRU SBA training and competency based assessment.

The critical analysis of clinical data though an audit cycle has proved useful in amending clinical practice in both high and low income countries [8,9]. Briefly, the audit cycle entails identifying the need for change, setting criteria to be met, collecting data on performance, assessing performance against the criteria and creating an action plan, then re-evaluating the performance at a set time frame after implementation of the action plan. At SMRU data for each pregnant woman is recorded routinely by SBAs and doctors. Birth records are double checked by doctors to ensure they are suitable for digital data entry. A WHO safe motherhood needs assessment in Maela camp has found very high accuracy of maternity record keeping [5].

In an audit of 2011 birth data, a high rate of nuchal cord ligation before the birth of the shoulders was observed: 15.3% (321/2097). Nuchal cord, the presence of one or more loops of umbilical cord wrapped around the neck of the fetus at birth, is common and can be expected in one in three to five labours [10-13]. In the two largest studies, nuchal cord was not associated with adverse perinatal outcome [10,14]. Furthermore tight nuchal cord, defined as inability to manually reduce the loop over the head, was recorded in 6.6% of 219,337 live births and was also not associated with adverse neonatal outcome [10]. The practice of umbilical cord ligation before birth stops the flow of blood between the baby and placenta and increases the risk of fetal morbidity and mortality from neonatal hypovolaemia [15-17], anaemia [18], and hypoxic-ischaemic encephalopathy particularly when birth is delayed by shoulder dystocia [19-21]. As nuchal cord is not associated with adverse perinatal outcome, potentially harmful birth techniques are important to rectify [10,14].

Approaches to nuchal cord practice result from what has been taught and learned from personal experience and from diffusion within the workplace [22]. Midwifery guidelines for management of nuchal cord are variable [11,16,23]. UK midwives reported everything from clamping and cutting of loose nuchal cords to hands-off approach for tight nuchal cords [22]. American College of nurse-midwives reported that just over half ( 57%) would clamp and cut the cord when it was very tight, 40% selected the somersault manoeuvre [24] and only 3.2% would clamp and cut the cord in most circumstances where a nuchal cord was present [25]. Checking for a nuchal cord is a common practice in the US, UK and Australia, but not in Norway and Denmark [26]. Vaginal examination in second stage to feel for nuchal cord is invasive, often painful and performed without informed consent [27,28] and scientific evidence to support its routine practice is lacking [26]. It was found that once midwives feel for a nuchal cord they revert to the intervention taught during their training: to clamp and cut the cord [28]. The practice of pulling loose loops of cord over the baby’s head also lacks evidence and may interfere with the normal physiology of birth and risks avulsion and subsequent neonatal bleeding [28].

Understanding the capacity and perceptions of SBAs working in resource limited settings can inform the pedagogy that best enhances safe practice with this cadre [29,30]. This manuscript aims to describe the use of the audit cycle on the practice of nuchal cord ligation, first by doctors, and then by a registered midwife [30,31]. In-depth interviews and a knowledge survey of SBAs were used to determine perceptions and knowledge about fetal nuchal cord and for SBAs to describe how a registered midwife influenced their practice.

## Methods

### Setting

SMRU is located in Tak Province and has clinics with delivery facilities situated on the Thai side of the border with Myanmar: a map of clinic locations has been published previously [4,32]. The first SMRU clinic is located in Maela (60 km north of Mae Sot), the largest refugee camp in Thailand with an estimated population of 45,000. Première Urgence – Aide Médicale Internationale is the major health provider in Maela. More than 90% of pregnant women in the camp attend antenatal care [32] and there are more than 1,200 deliveries per year in the birth facility. Services for migrants commenced in 1998 and the population served is estimated at 200,000. SMRU also has two birth facilities for migrants, the first of which opened at Wang Pha (30 km north of Mae Sot) in December 2007 and the second at Maw Ker Thai (65 km south of Mae Sot) in April 2010, which together do more than 1,100 deliveries per year.

Local housing in both refugee and migrant communities is limited to bamboo or concrete walls and flooring with leaf or metal roofing and pit toilets. Water must be carried to the house from communal wells and electricity is usually not available. Basic education is supported by non-government organizations in the camp and provided by community organizations in the migrants. Quality of education is not formally assessed and certificates achieved at these schools are not recognized by the Thai or Myanmar governments. Minimal educational opportunities exist after the age of sixteen. Basic rations are provided to refugees who hold a UNHCR registration card. Migrants usually work for a daily wage and do not receive any assistance with food supplies. In both migrant and refugee settings acute malnutrition is rare but vitamin deficiencies have been a problem within refugee camps, thiamine (vitamin B1) in particular [33].

### SMRU maternity services

Maternity services provided by SMRU includes: antenatal care (routine investigation and treatment of malaria, anaemia and HIV, ultrasound examination, vitamin supplements, vaccination, health information and advice, care of medical/obstetric complications), care in labour and childbirth, and post-partum care. Eight of the nine ‘signal functions’ of SBA including parenteral administration of an oxytocic, antibiotic and anticonvulsant, manual removal of retained placenta, removal of retained products of conception, assisted delivery, resuscitation of a baby using a bag and mask, and blood transfusion can be provided on site [34]. Caesarean section cases are referred by a 24 hour stand-by car to the nearest Thai hospitals: 30 to 75 minutes from the clinics. There has been a significant shift in this border region from traditional birth attendant home deliveries to SBA deliveries in SMRU birth facilities [4,32]. Amongst women who attend SMRU antenatal care, more than 80% deliver at SMRU. The ‘SBA-led’ unit is open 24-hours. Registered doctors with non-specialist obstetric qualifications are present Monday to Friday and support the SBAS by telephone after hours and on the weekends.

Since 1998 the unit has used evidenced-based clinical protocols for obstetric and medical practice. The first version of the document was produced in three languages (Karen, Burmese and English) and the latest version of the SMRU guideline is only available in English. Diagrams are provided when possible and the section on nuchal cord includes an image of clamping with scissors in place to cut a double looped, tight nuchal cord.

### Study design

**Figure 1:**
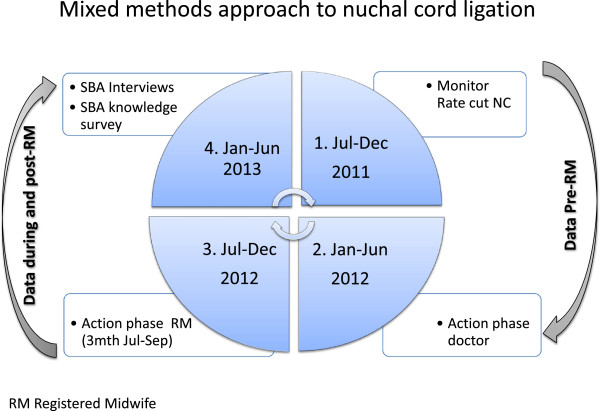
**Timing of audit cycle events.** RM- registered midwife.

A mixed methods approach [35] was undertaken. Clinical audit data analysis was completed to monitor the effectiveness of the intended practice change by examining rates of nuchal cord ligation with instruction from doctors, with the presence of a registered midwife, and in the period up to 9 months after departure of the registered midwife. Interviews were then held with the SBAs in an attempt to identify why SBAs wanted to clamp and cut the cord and how the registered midwife helped them change their practice. A knowledge survey was also undertaken to verify what linkage SBAs made between knowledge and practice. The approximate timing of the audit cycle events and other study data collection points are presented in Figure 1.

### Action phase of the audit cycle

In Jan-2012, after reviewing data from the last two quarters of 2011, the doctors were given responsibility to reduce high rates of cord ligation at each birth facility. No formal lectures were done, the problem was explained to the staff at the regular midwife meetings and correct practice was encouraged whenever the doctor was in the delivery room. In general the doctor was mostly there for problems, not routine deliveries. All of the doctors can speak Burmese or Karen so language barriers are minimal. The audit cycle in June 2012 revealed this approach had a limited impact on the rate of nuchal cord ligation.

The next attempt was implemented by an experienced registered midwife. The registered midwife originally undertook hospital based training in Scotland and later completed a graduate diploma in Midwifery and a Master of Midwifery in Australia. MP has a total of 24 years work experience in UK and in Australia, predominantly in birthing, across rural and tertiary settings that have also involved mentoring junior registered midwives although not explicitly employment as an educator. This was her first experience of working with SBA in a resource limited setting.

During a week of observing the birth techniques of SBAs, the practice of routinely checking for nuchal cord and ligation before the birth of the shoulders was confirmed and identified as a problem. The literature was reviewed and suggested changes and advice for SBAs included:

1. No routine checking for nuchal cord. This was based on the tendency to clamp and cut if nuchal cord was identified and supported the fact that nuchal cord is normal and doesn’t require routine intervention [10].

2. Only clamping and cutting a nuchal cord if it was preventing the birth from occurring.

3. Rewriting of the current SMRU guideline for management of fetal nuchal cord.

In conjunction with providing clinical support in the birth room at Maela, brief education sessions were also performed. The registered midwife identified that education sessions were ineffective in changing practice, in part due to the language barrier as English was often a second, third or fourth language for the SBAs.

The registered midwife was available ten hours a day, six days per week in Maela birth facility which was staffed by a total number of twenty six SBAs including six senior SBAs. At each birth with a fetal nuchal cord, preservation of umbilical cord circulation was the aim. Being present at the birth allowed the registered midwife to encourage and support the SBAs to facilitate birth with the nuchal cord intact. On observing the presence of nuchal cord the SBA often sought assistance with cries of “cord, cord”. Reassurance from the registered midwife that this was “no problem” and encouragement to continue to allow the birth to proceed without cutting and clamping the cord was all that was required. Following the birth the cord was gently unwrapped from the baby’s neck and/or body. Three SBAs were often present during birth and this provided them the opportunity to observe the management of birth with nuchal cord left intact. Those who had gained experience in facilitating birth with intact nuchal cord then supported and reassured their colleagues. The senior SBAs rotated from Maela to the Maw Ker Thai site. In September the registered midwife spent three days at Wang Pha clinic where she explained evidence-based management of nuchal cord and showed a short video of a delivery taken in Maela. There were no deliveries with nuchal cord during this short period at Wang Pha.

### Semi-structured interviews with SBA

Interviews of SBAs were conducted on a one to one basis by an independent researcher (CPD) familiar with the camp and clinic situation; fluent in written and spoken Karen, Burmese and English language and holding a Bachelor of Education. Interviews were carried out on one day in Maela camp. The purpose of the interview was to gain an understanding of why SBAs wanted to clamp and cut the cord and how “Theramu” (traditional and respectful term used to name a teacher) helped them change their practice more than the site doctor.

The interviews were conducted in a private area near the clinic on a single day in mid- February 2012. Five SBA were asked if they agreed to be interviewed and none refused. While the senior SBA was interviewed first, subsequent interviewees were chosen in turn by the SBA who had just been interviewed. Notes were taken during the interview in the original language: Karen, Burmese and/or English (based on interviewee preference) and later translated into English by the interviewer.

The interviews were semi-structured and started with a fixed statement aimed at putting the SBA at ease: “Today I want to give you some good news and ask you about the improvement of SBA practice in the delivery room. We want to understand more about the improvement in delivery of the baby with cord around the neck. In the past the doctor tried to explain not to rush to cut the cord on the perineum but was not successful. When Theramu came only for three months here you improved a lot.” This was followed by a question: “How did Theramu explain or teach you?” Depending on the response and flow of conversation any of the following questions were asked: How did Theramu make it easy for you to understand? Was it explained to you that it was dangerous to clamp and cut the cord on the neck? Did Theramu do the delivery herself? How did Theramu show you the correct way to do it? Would you like Theramu to teach you more about delivery?

The interviewer and a doctor with more than 19 years of experience with SBA in this population read the English transcript of the interviews. The transcript was printed in duplicate and cut-up into sections by statement so that the statements could be sorted into meaning units, independently by the interviewee and doctor [36]. Two major themes emerged in relation to the change in practice and disagreements about statement placement were resolved by discussion. Both interviewer and doctor recognized snippets within the aforementioned statements referring to pedagogy and it was agreed to highlight these independently.

### Knowledge survey

A survey of basic knowledge and practice of nuchal cord management was conducted the day after the in-depth interviews. Knowledge and practice questions were fixed answer (multiple choice or true/false) and if the answer to one question (Is nuchal cord harmful to the infant?) was yes, they were asked to explain why. An additional question assessed potential language barriers between SBAs and the registered midwife. When the survey was completed the SBAs were shown a 12 slide power-point presentation which emphasized the good change in practice. Overall there were 26 SBAs who were surveyed including 6 senior, 8 junior and 12 assistants with a period of employment in the birth centre, of 6 [4-9], 3 [2-15] and 2 [1,2] years.

### Data extraction

All birth records from 1-July-2011 to 30-June-2013 were extracted and analyzed using SPSS v20.0. Proportions were compared using the chi-squared test, normally distributed data was compared with Student’s t-test and parametric data using the Mann–Whitney U test.

### Ethical statement

The data presented in this manuscript was approved for use by the Oxford Tropical Research Ethics Committee (OXTREC: 28–09, amended 19-April-2012) and by the local Tak Province Border Community Ethics Advisory Board. Verbal consent was obtained for in-depth interview as no risk was involved for the SBA and participation was voluntary. All responses have been de-identified and names have been changed to ensure confidentiality.

## Results

### Rates of umbilical cord ligation

**Figure 2:**
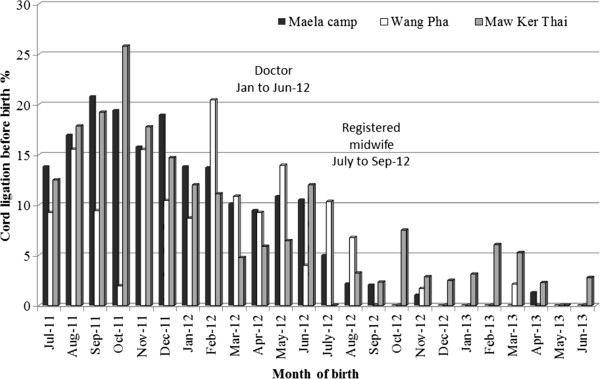
**Rates of umbilical cord ligation before delivery during the audit cycle.** Rates were monitored at the three birth units of Shoklo Malaria Research Unit and included: MKT - Maw Ker Thai (migrant site), MLA – Maela (refugee camp) and WPA – Wang Pha (migrant site).

The rate of cord ligation before birth was calculated for 4,270 normal singletons that delivered with a gestational age of ≥28 weeks over a 24 month period (Figure 2). When the two year period was examined by quarters there was a significant linear trend (p < 0.001) for decreasing rates of cord ligation before birth: 15.9% (178/1123) vs 11.1% (107/966) vs 2.4% (28/1182) vs 0.9% (9/999), from quarter one through four, respectively. The registered midwife departed half-way through the third quarter. The difference in rates between the first and second quarter under the supervision of the doctors was significant (p = 0.002) but a much greater reduction was observed between the second and third quarter with the registered midwife. In addition there was a significant decrease in the proportion of cord ligation rates at each site with a birth facility before (1-Jul-2011 to 30-Jun-12) and after (1-July-12 to to 30-Jun-13) the arrival of the registered midwife: Maela 15.3% (172/1127) vs 1.0% (10/1045); Wang Pha 10.6% (65/611) vs 1.9% (13/675) and Maw Ker Tai 13.7% (48/351) vs 3.0% (14/461), p < 0.0001 for all comparisons. This occurred at all sites while the registered midwife was primarily based in Maela for three months. Other sites were exposed to the concepts taught in Maela through the registered midwife’s three-day visit to Wang Pha at the end of August 2012, and through rotation of senior SBAs between sites.

The rates of stillbirth amongst normal singletons was not significantly different between the two 12 month periods: 0.5% (11/2089) vs 0.9% (19/2181), p = 0.202. Amongst the live born, normal singletons there was no significant difference in birth weight or gestational age, or proportion with shoulder dystocia; which was very low in this population (Table 1) The median Apgar scores at one and five minutes were statistically significantly higher after the three month presence of the registered midwife, although this did not change the median score (Table 1). There was no significant difference in the proportion of five minutes Apgar scores less than 7 (Table 1) and the difference in one and five minute Apgar scores came from a shift in the proportion of newborns assigned a score of nine at one minute and ten at five minutes (data not shown). A lower proportion of newborns required resuscitation and a lower proportion of mothers had post-partum haemorrhage but no difference was observed in the rate of episiotomy (Table 1).

**Table 1:** Outcomes for live born normal singletons and their mother prior and after a three month registered midwife placement

**Outcome measured**	**One year pre-arrival of registered midwife**	**One year during and post arrival registered midwife**	**p value**
	**n = 2078**	**n = 2161**	
Liveborn, normal, singletons	n = 2078	n = 2162	
Birthweight, g	2979 ± 437 [1120–4480]	2989 ± 429 [710–4810]	0.422
Mean estimated gestation age, wks	39.1 ± 1.5 [28.1-43.4]	39.1 ± 1.4 [28.1-42.0]	0.701
Shoulder dystocia, % (n)	0.1% (2/2078*)	0.1% (3/2162)	1.000
Apgar^1^, median	9 [1-10], n = 2067	9 [1-10], n = 2148	0.004
Apgar^5^, median	10 [1-10], n = 2067	10 [1-10], n = 2148	0.002
Proportion Apgar^5^ <7, % (n)	0.8% (17/2067)	0.8% (18/2148)	1.000
Newborn resuscitation, % (n)	2.8% (59/2071)	1.6% (34/2162)	0.006
Post-partum haemorrhage, % (n)	8.3% (172/1904)	6.5% (141/2162)	0.030
Episiotomy, % (n)	5.1 (105/2048)	5.2 (110/2127)	1.000

Missing data: birth weight and gestation and shoulder dystocia n = 0; Apgar^1^ = 25; Apgar^5^ = 25; newborn resuscitation n = 6; post-partum haemorrhage n = 2; Episiotomy n = 65.

*one of whom had cord ligation before delivery of the shoulders (EGA 39.5wks weight 4380 7^1^ 9^5^).

### Semi-structured interviews with SBA

The SBAs in Maela related their change in practice within two major themes: i. a strong belief associated with fear and worry, that the fetus with nuchal cord would not be able to breath, and ii. confidence in their own ability to allow normal delivery of these infants when supported by a registered midwife. These themes are demonstrated in the following statements:

“We afraid it [the nuchal cord] is too tight and baby can’t breathe so we clamp and cut; we afraid we will have to do resuscitation if clamp and cut”.

“Before Theramu was here whenever we have cord tangle [d] on [the] neck we cut because we were worried, we [were] concern [ed] for the baby. When she was here she explained us, [there is] no need to worry. She make us wait until baby out and sometime undo the cord by the upper part of the head or sometime lower [or] sometime loosen it around the body of the baby after baby out. When it gets loose [end] then proceed with the delivery. She explain [ed] baby [ies] can come out by themselves. The only thing is to wait and loosen the cord. Before she was here, the reason why we cut the cord is because we were too worried. We afraid baby won’t be able to breathe”.

“Theramu did not tell us it is dangerous to cut the cord on the neck but explained us if we deliver the women this way, the right way, it is not harmful for the baby… to wait and undo the cord is correct and no need to worry”.

“Theramu observes us for one week, then she [did] not do [delivery], only tell [told] us what is the right way to do when baby is coming out”.

“Theramu observe [d] us, and explain [ed to] us as we do delivery, so we see we can do it, and we surprised because it was so easy. She has patience with us”.

“When Theramu deliver [ed] with us we are afraid when the cord is on the fetus neck, we fear the baby won’t have enough oxygen, Theramu is not afraid, Theramu make us not afraid [too], when Theramu is by my side I have more confidence”.

Specific characteristics of the registered midwife’s pedagogic approach were melded into many of the aforementioned statements. These were thought to be significant when reflecting on the higher rate of improved nuchal cord practice in comparison to the rate achieved by the doctors. These include “she did not do”; “only explained/only tell”; “by my side”; “make us not afraid”; “has patience with us” and “did not tell us it was dangerous”.

### Knowledge survey results

The survey was conducted three months after the departure of the registered midwife. The results of the knowledge survey (Table 2) show that most SBAs know the proportions of infants born with nuchal cord and that is can be associated with fetal distress in the second stage of labour. The majority answered the question about harm from fetal nuchal cord incorrectly. The reason for harm in the 20/24 (83.3%) who provided one included low fetal oxygen 16/20 (80.0%) and fetal distress 4/16 (20.0%). The dangers associated with ligation of the umbilical artery circulation are not completely understood, except in association with shoulder dystocia (Table 2). When asked about current practice of a loose nuchal cord only one SBA reported they would ligate the umbilical artery before delivery of the body 1/26 (3.3%); while this was 21/26 (80.8%) if the nuchal cord was tight.

**Table 2:** Proportion of correct answers given by skilled birth attendants according to current position at SMRU

**Question**	**Senior**	**Junior**	**Assistant**	**Total**
Proportion of infants born with nuchal cord	66.7 (4/6)	75.0 (6/8)	50.0 (6/6)	61.5(16/26)
Presence of fetal nuchal cord is harmful to the infant	16.7(1/6)	0 (0 of 8)	8.3 (1/11)	16.7 (2/26)
Can cause fetal distress in 2^nd^ labour	100.0 (6/6)	87.5 (6/6)	91.7 (11/12)	92.3 (24/26)
**Clamping and cutting of the fetal nuchal cord before birth:**				
…is a dangerous practice	83.3 (5/6)	100.0 (8/8)	75.0 (9/12)	84.6 (22/26)
…associated with fetal anaemia	83.3 (5/6)	87.5 (7/8)	75.0 (9/12)	83.3 (21/26)
…associated with fetal shock and hypovolaemia	50.0 (3/6)	25.0 (2/8)	41.7 (5/12)	38.5 (10/26)
…associated with HIE especially with shoulder dystocia	100.0 (6/6)	100.0 (8/8)	75.0 (9/12)	88.5 (23/26)
Overall score, %	71.4	62.5	57.1	62.1

HIE-hypoxic ischaemic encephalopathy.

All SBAs reported they understood some, not all, of what they heard in English from the registered midwife.

## Discussion

Despite language differences a three month presence of a registered midwife had a profound impact on the rate of umbilical cord ligation before birth for fetal nuchal cord amongst SBAs. High rates of good practice started to occur once the registered midwife supported SBA in the birth facility and good practice rates were maintained well after the departure of the registered midwife. This change occurred despite SBAs incorrect knowledge about nuchal cord. Most SBAs in this setting thought fetal nuchal cord was harmful contrary to published evidence and their appreciation of the possible harm of umbilical cord ligation was incomplete [10].

The audit cycle used here was a useful model for change [30,31]. The steady presence of the registered midwife led to a greater reduction in cord ligation before birth than with the doctor. When exploring why this was so the interviews suggest the pedagogic approach of the registered midwife that included presence during normal births, support at the critical time of labour, non-threatening interaction, guidance and reassurance not to cut when nuchal cord was observed, and patience were key elements in success. Clearly the relationship established by the registered midwife with the SBA was conducive to experiential learning [37]. Asian culture respects age and wisdom and in rural areas this tradition remains deeply entrenched within the population and while this was not highlighted by the SBAs it is very likely to have favoured a positive relationship for the experienced registered midwife [38]. The interviews suggested the registered midwife imparted confidence to the SBA in their own ability to deliver. This confidence is reflected in the changes in ligation rates from senior SBAs who rotated to other SMRU clinics who had the confidence to teach and implement positive change with colleagues without the registered midwife presence [39]. The contrary finding of incorrect knowledge and good clinical performance amongst the SBA in this setting has been observed even in countries with tertiary qualified staff [40]. We have observed but not documented until now that training SBAs in this setting is best done by practical and hands-on training. This observation has tremendous significance in skilling of SBAs in similar areas where barriers to tertiary level education exist [41].

One of the limitations of this manuscript is that no attempt was made to identify how the practice of cutting the nuchal cord reached high levels. One of the possibilities was that in the local obstetric guideline, if it is not read, the most obvious way to manage the problem comes from the diagram demonstrating ligation of nuchal cord. The SMRU obstetric guideline is currently being updated and diagrams, originally placed in the document to enhance understanding are now being discussed with SBAs to ensure they are helpful and not misleading bearing in mind the limitations on literacy in English. The changes in birth outcomes have not been controlled for in any way and other training that occurred over the lengthy time period of the survey may explain the changes. Nevertheless the reduction in rates of cord ligation before birth has not been associated with detrimental effects on birth outcome.

The registered midwife remained at Maela throughout the action phase of the audit cycle period apart from a three day visit to Wang Pha. The furthest site from Maela Refugee camp was the slowest to change practice as this relied soley on the rotation (once every six weeks) of the senior SBA to that site, nevertheless positive change has occurred although it has not been as successful as Maela and Wang Pha.

## Conclusions

The data presented here demonstrates that a supportive, registered midwife provided appropriate mentorship and feedback to SBAs on a targeted birthing skill. SMRU will continue to encourage interprofessional education of SBAs particularly from experienced registered midwives on evidence based practice in birth facilities. The benefit of basic audit and registered midwife interprofessional learning could be used in other health structures where SBAs provide care in childbirth in resource limited settings

## Competing interests

The authors declare they have no competing interests.

## Authors’ contributions

MP, NSW, PSS, MN, NWT, AM and MEG carried out the steps of the audit cycle. NSW, PSS, MN, NWT, AM, MEG participated in birth site data collection. CPD carried out the in-depth interviews and CP and RM participated in their thematic analysis. CP carried out the knowledge survey. MP, NWT, AM, MEG participated in the design of the study and RM performed the statistical analysis. MEG, AM, NWT, RM and FN conceived of the study, and participated in its design and coordination and helped to draft the manuscript. All authors read and approved the final manuscript.

## Pre-publication history

The pre-publication history for this paper can be accessed here:

http://www.biomedcentral.com/1471-2393/14/76/prepub
